# Supplementary material for: Assessment of Heavy Metals Contamination and Antimicrobial Drugs Residue in Broiler Edible Tissues in Bangladesh
Source: Antibiotics (Basel). 2023 Mar 28;12(4):662. doi: 10.3390/antibiotics12040662 (PMC10135153; doi:10.3390/antibiotics12040662)
Supplement: Supplementary file 1 [file antibiotics-12-00662-s001.zip › antibiotics-2297530-supplementary.pdf]

Assessment of heavy metals contamination and antimicrobial drugs residue in broiler edible tissues in Bangladesh

Shaikh Mohammad Bakhtiar[1,†], Mohammad Rafiqul Islam[2,†], Md. Jisan Ahmed[3], Abdur Rahman[4], Kazi Rafiq[5, †,*]

[1]Bangladesh Agricultural Research Council (BARC), Dhaka 1215, Bangladesh

[2]Livestock Division, Bangladesh Agricultural Research Council (BARC), Dhaka 1215, Bangladesh

[3]Department of Pathology, Faculty of Animal Science and Veterinary Medicine, Sher-e-Bangla Agricultural University, Dhaka-1207, Bangladesh

[4]Dept. of Dairy Science, Faculty of Animal Science and Veterinary Medicine, Sher-e-Bangla Agricultural University, Dhaka-1207, Bangladesh

[5]Department of Pharmacology, Bangladesh Agricultural University, Mymensingh 2202, Bangladesh

***Correspondence:** kazirafiq@bau.edu.bd; Tel.: +880-1711-285766; Fax.: +880-916-1510

†These authors contributed equally to this work.

Supplementary Methods and materials:

Study area

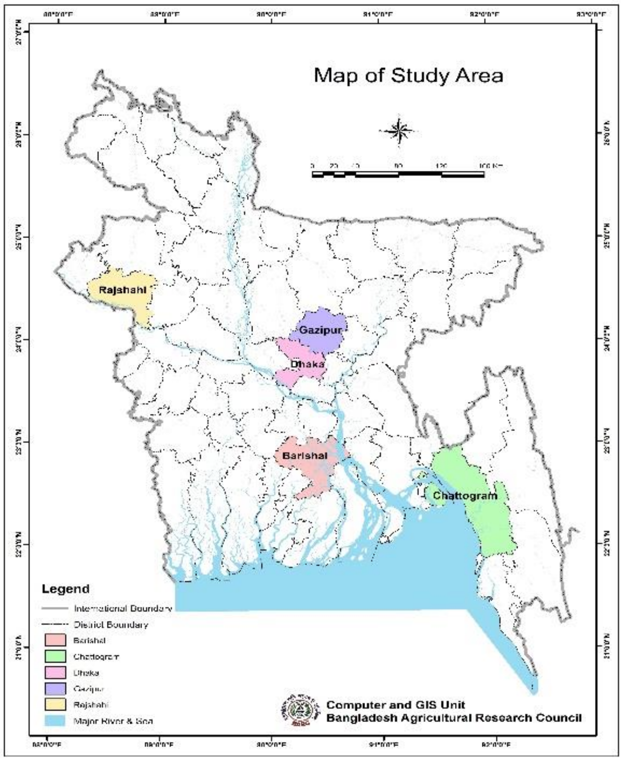

Supplementary Figure S1. Map of study areas: Dhaka, Chattogram, Gazipur, Rajshahi, and Barisal districts of Bangladesh.

“Qualitative and Quantitative Study of Antibiotic and Heavy Metal Residues in Broiler” প্রকল্প

প্রাণিসম্পদ বিভাগ

বাংলাদেশ কৃষি গবেষণা কাউন্সিল

ফার্মগেট, ঢাকা-১২১৫

১। খামারের নামঃ

২। খামারীর নামঃ

৩। ঠিকানাঃ

৪। মোবাইল নম্বরঃ

৫। খামারে ব্রয়লারের সংখ্যাঃ

৬। আপনি ব্রয়লারকে কি ধরনের খাবার খাওয়ান? ক) রেডি ফিড খ) খামারের বানানো

৭। ব্রয়লার খাবার বা পানির সাথে কি ভিটামিন/মিনারেল প্রিমিক্স মেশান? হ্যাঁ / না

৮। ব্রয়লার খাবার বা পানির সাথে কি এন্টিবায়োটিক মেশান? হ্যাঁ / না

- যদি হ্যাঁ হয়, তবে কি এন্টিবায়োটিক মেশান?
- কত দিন বয়সের ব্রয়লারকে এন্টিবায়োটিক মেশানো খাবার বা পানি দেন?
  - ০-৭ / ৮-১৪ / ১৫-২১ / ২১-২৮ / ২৯ দিন এর উপর

৯। ব্রয়লার অসুস্থ হওয়ার আগে কি এন্টিবায়োটিক খাওয়ান?

- যদি হ্যাঁ হয়, তবে কি এন্টিবায়োটিক খাওয়ান?

১০। আপনি কি এন্টিবায়োটিক ব্যবহারের ক্ষতিকর দিক সম্পর্কে জানেন? ? হ্যাঁ / না

১১। আপনি কি এন্টিবায়োটিক এর প্রত্যাহার কাল সম্পর্কে জানেন? ? হ্যাঁ / না

১২। আপনি কি এন্টিবায়োটিক এর রেসিডিউ সম্পর্কে জানেন? ? হ্যাঁ / না

<u>ধন্যবাদ</u>

“Qualitative and Quantitative Study of Antibiotic and Heavy Metal Residues in Broiler” প্রকল্প

প্রাণিসম্পদ বিভাগ

বাংলাদেশ কৃষি গবেষণা কাউন্সিল

ফার্মগেট, ঢাকা-১২১৫

১। নামঃ | ২। পুরুষ /মহিলা

৩। পেশাঃ | ৪। শিক্ষাগত যোগ্যতাঃ

৫। ঠিকানাঃ | ৬। মোবাইল নম্বরঃ

৭। আপনি কি ব্রয়লার মাংস খান? হ্যাঁ / না

যদি খান, তাহলে কেন খান?

যদি না খান, তাহলে কেন খান না?

৮। সপ্তাহে কয় দিন ব্রয়লার মাংস খান?

৯। ব্রয়লার মাংস কিভাবে খান? রান্না করে / গ্রীল করে / ফ্রাই করে / অন্য উপায়ে

১০। ব্রয়লার মাংস খাওয়ার কি কি উপকারিতা আছে?

১১। ব্রয়লার মাংসের কোন ক্ষতিকর দিক আছে কি না? হ্যাঁ / না

কি কি ক্ষতিকর দিক আছে?

১২। ব্রয়লার মাংস উৎপাদনে কি এন্টিবায়োটিক ব্যবহার করা হয়? হ্যাঁ / না

১৩। ব্রয়লার মাংস উৎপাদনে কি হরমোন ব্যবহার করা হয়? হ্যাঁ / না

১৪। ব্রয়লার মাংসে কি ভিটামিন, আমিষ থাকে? হ্যাঁ / না

১৫। ব্রয়লার মাংস উৎপাদনে কি ক্ষতিকর এন্টিবায়োটিক/হরমোন থাকে? হ্যাঁ / না

১৬। ব্রয়লার মাংস খেলে কি রোগ প্রতিরোধ ক্ষমতা বাড়ে? হ্যাঁ / না

১৭। ব্রয়লার মাংস খেলে কি করোনা ভাইরাস প্রতিরোধ করা যেতে পারে? হ্যাঁ / না

Supplementary Results:

Supplementary Table S1: Overall Correlation among antimicrobials and Heavy metals

**Antimicrobial/ Heavy Metal**		**Arsenic**	**Chromium**	**Lead**
Sulfadiazine	Pearson Correlation	**0.114***	-0.046	-0.067
	Sig. (2-tailed)	0.050	0.437	0.255
	n	294	294	294
Arsenic	Pearson Correlation	1	**-0.123***	**-0.203****
	Sig. (2-tailed)		0.035	0.000
	n	295	295	295
Chromium	Pearson Correlation	-0.123*	1	**0.341****
	Sig. (2-tailed)	0.035		0.000
	n	295	295	295

Supplementary Table S2: Correlation among antimicrobials and Heavy metals found in Broiler meat

**Antimicrobial/ Heavy Metal**		**Arsenic**	**Chromium**	**Lead**
Sulphadiazine	Pearson Correlation	**0.114***	-0.046	-0.067
	Sig. (2-tailed)	0.050	0.437	0.255
	n	294	294	294
Arsenic	Pearson Correlation	1	**-0.123***	**-0.203****
	Sig. (2-tailed)		0.035	0.000
	n	295	295	295
Chromium	Pearson Correlation	-0.123*	1	**0.341****
	Sig. (2-tailed)	0.035		0.000
	n	295	295	295

Supplementary Table S3: Correlation among antimicrobials and Heavy metals found in Broiler meat in Barisal

Antimicrobial/ Heavy Metal		Arsenic	Chromium	Lead
Oxytetracycline	Pearson Correlation	-0.155	0.007	**0.286***
	Sig. (2-tailed)	0.262	0.962	0.036
	n	54	54	54
Doxycycline	Pearson Correlation	**-0.320***	0.073	**0.348****
	Sig. (2-tailed)	0.018	0.598	0.010
	n	54	54	54
Sulphadiazine	Pearson Correlation	**-0.308***	0.011	0.058
	Sig. (2-tailed)	0.025	0.940	0.678
	n	53	53	53

Supplementary Table S4: Correlation among antimicrobials and Heavy metals found in Broiler meat in Dhaka

Antimicrobial/ Heavy Metal		Chloramphenicol	Oxytetracycline	Sulphadiazine	Doxycycline	Chromium
Arsenic	Pearson Correlation	-0.070	**0.237***	**0.275***	-0.128	-0.136
	Sig. (2-tailed)	0.562	0.045	0.019	0.284	0.254
	n	72	72	72	72	72
Lead	Pearson Correlation	**0.249***	-0.024	-0.069	**0.245***	**0.462****
	Sig. (2-tailed)	0.035	0.840	0.565	0.038	0.000
	n	72	72	72	72	72

Supplementary Table S5: Correlation among antimicrobials and Heavy metals found in Broiler meat in Gazipur

Antimicrobial/ Heavy Metal		Arsenic	Lead
Sulphadiazine	Pearson Correlation	**0.355***	0.182
	Sig. (2-tailed)	0.013	0.216
	n	48	48
Chromium	Pearson Correlation	-0.203	**0.400****
	Sig. (2-tailed)	0.167	0.005
	n	48	48

Supplementary Table S6: Correlation among antimicrobials and Heavy metals found in Broiler meat in Rajshahi

Heavy Metal		Lead
Chromium	Pearson Correlation	**0.365****
	Sig. (2-tailed)	0.003
	n	66

Supplementary Table S7: Correlation among antimicrobials and Heavy metals found in Broiler meat in Large Farm

Heavy Metal		Lead
Arsenic	Pearson Correlation	**-0.356****
	Sig. (2-tailed)	0.004
	n	63
Chromium	Pearson Correlation	**0.524****
	Sig. (2-tailed)	0.000
	n	63

Supplementary Table S8: Correlation among antimicrobials and Heavy metals found in Broiler meat in Medium Farm

Heavy Metal		Chromium
Arsenic	Pearson Correlation	**-0.263***
	Sig. (2-tailed)	0.038
	n	63

Supplementary Table S9: Correlation among antimicrobials and Heavy metals found in Broiler meat in Small Farm

**Heavy Metal**		**Lead**
Chromium	Pearson Correlation	**0.416****
	Sig. (2-tailed)	0.001
	n	63

Supplementary Table S10: Correlation among antimicrobials and Heavy metals found in Broiler meat in Market

**Heavy Metal**		**Lead**
Chromium	Pearson Correlation	**0.312****
	Sig. (2-tailed)	0.003
	n	88
